# Correlation of *TP53* Genetic Alterations with p53 Immunohistochemical Expression and Their Prognostic Significance in DLBCL

**DOI:** 10.3390/curroncol32090488

**Published:** 2025-08-31

**Authors:** Chen Chen, Zijuan Hu, Min Ren, Longlong Bao, Ran Wei, Tian Tian, Xiaoli Zhu, Qianming Bai, Baohua Yu, Xiaoqiu Li, Xiaoyan Zhou

**Affiliations:** Department of Pathology, Fudan University Shanghai Cancer Center, Shanghai 200032, China; chenc22@m.fudan.edu.cn (C.C.); huzj23@m.fudan.edu.cn (Z.H.); 21111230072@m.fudan.edu.cn (M.R.); bll122011648@126.com (L.B.); rwei17@fudan.edu.cn (R.W.); tiantian2016922@163.com (T.T.); zhuxl@shca.org.cn (X.Z.); baiqianming@shca.org.cn (Q.B.); yubh2014@163.com (B.Y.); leexiaoqiu@hotmail.com (X.L.)

**Keywords:** diffuse large B-cell lymphoma, *TP53* mutations, next-generation sequencing, copy number variation, p53 immunohistochemistry, prognostic markers

## Abstract

*TP53* genetic alterations represent a critical molecular feature in diffuse large B-cell lymphoma (DLBCL), with well-established associations with aggressive disease behavior and therapeutic resistance. However, significant controversy persists regarding the clinical utility of p53 immunohistochemical (IHC) expression as a surrogate marker. This study presents a thorough investigation of *TP53* genetic alterations and their correlation with p53 protein expression in 664 cases of DLBCL. Using targeted next-generation sequencing (tNGS), we identified *TP53* alterations (mutations and/or copy number losses (CNLs)) in 170 cases (25.6%). Among them, 161 cases had mutations. Concurrent analysis of copy number variations (CNVs) in 109 cases revealed *TP53* CNLs in 17.4% (19/109), with 68.4% (13/19) of these showing coexisting mutations. Immunohistochemical evaluation of p53 expression in 371 cases demonstrated strong positivity (≥65% cells) in 21% (78/371), complete negativity (<1%) in 5.7% (21/371), and wild-type pattern (1–65%) in 73.3% (272/371) of cases. The p53 IHC laboratory-developed test (LDT) showed 79.2% sensitivity and 91.6% specificity for detecting *TP53* alterations overall, though sensitivity varied significantly by mutation type: 86.2% for missense mutations but only 14.3% for nonsense mutations. Clinically, cases with *TP53* alterations exhibited more aggressive disease characteristics, including higher ECOG performance scores, increased frequency of B symptoms, and poorer initial treatment responses (complete response rate 68.3% vs. 82.5% in wild-type cases). Most importantly, *TP53* genetic alterations, but not p53 protein expression patterns, emerged as an independent prognostic factor for progression-free survival. Our findings demonstrate that tNGS effectively identifies most *TP53* alterations and complementary CNV analysis enhances detection of copy number losses. The p53 IHC LDT serves as a useful but imperfect screening tool, with high specificity but variable sensitivity depending on mutation types. These results have important implications for molecular diagnostics in DLBCL, supporting the necessity for comprehensive genetic testing rather than reliance on protein expression analysis alone for accurate risk stratification and treatment planning.

## Simple Summary

*TP53* genetic alterations represent well-established prognostic markers in diffuse large B-cell lymphoma (DLBCL), consistently associated with aggressive clinical behavior and inferior outcomes. Next-generation sequencing (NGS) provides accurate detection of *TP53* mutations and is carried out widely in clinical practice, however, this methodology may exhibit limitations in identifying copy number variations (CNVs) and presents challenges in clinical implementation due to cost and technical requirements. This comprehensive study evaluated 664 DLBCL cases to: (1) assess NGS detection ability for *TP53* copy number losses (CNLs) and (2) investigate the diagnostic efficiency of p53 immunohistochemistry (IHC) as a potential surrogate marker for *TP53* genetic alterations. Our findings demonstrate that NGS successfully identified the majority of cases with *TP53* copy number alterations, which often occur simultaneously with mutations. Furthermore, while laboratory-developed test (LDT) for p53 IHC used in this study showed reasonable sensitivity for specific mutation subtypes (particularly missense variants), its performance was suboptimal for other genomic alterations. Importantly, only *TP53* genetic alterations emerged as a consistent predictor of survival outcomes. These results underscore the clinical necessity of integrating comprehensive genetic profiling with IHC analysis to optimize risk stratification and therapeutic decision making in DLBCL management.

## 1. Introduction

The *TP53* tumor suppressor gene is a critical regulator of cellular homeostasis and plays a central role in maintaining genomic stability [1,2]. Loss of *TP53* function due to mutations or copy number alterations (CNAs) is a hallmark of cancer, contributing to tumor initiation, progression, and therapeutic resistance [3]. *TP53* genetic alterations represent one of the most frequent genetic events in human malignancies, occurring in more than 50% of all cancers, including hematologic malignancies such as diffuse large B-cell lymphoma (DLBCL) [4]. In DLBCL, *TP53* mutations and copy number losses (CNLs) occur in approximately 20–30% of cases and correlate with aggressive clinical behavior, poor response to standard therapies (e.g., R-CHOP), and inferior overall survival [5,6,7,8,9,10]. These findings highlight the significance of *TP53* as a potential biomarker for risk stratification and therapeutic decision making in DLBCL.

*TP53* encodes p53, a transcription factor that governs critical cellular pathways including cell cycle arrest, apoptosis, senescence, and DNA repair in response to stress signals [11]. Under normal physiological conditions, p53 protein is maintained at low intracellular levels via MDM2-mediated ubiquitination and proteasomal degradation [12]. However, upon DNA damage or oncogenic stress, p53 becomes stabilized and activated through phosphorylation and acetylation, thereby inducing its target genes to maintain genomic integrity and prevent malignant transformation [2,11,12].

*TP53* genetic alterations include missense mutations, nonsense mutations, frameshift mutations, small insertions/deletions, splice-site mutations, and copy number losses [4]. Recent advances in molecular diagnostics, such as targeted next-generation sequencing (tNGS) and high-resolution copy number analysis (e.g., OncoScan array), have markedly improved the detection of *TP53* genetic alterations. NGS enables comprehensive identification of point mutations, indels, and splice variants [10,13], while the OncoScan array provides sensitive detection of copy number losses.

The biological consequences of these alterations are diverse. Missense mutations often produce a stable but dysfunctional protein with dominant-negative or gain-of-function properties, promoting cell survival, chemoresistance, and tumor progression. In contrast, nonsense and frameshift mutations usually result in truncated proteins leading to loss of tumor suppressor function [14,15,16,17]. Copy number losses cause reduced *TP53* gene dosage, further impairing p53-mediated tumor suppression [18]. Therefore, accurate detection and classification of *TP53* alterations are crucial for understanding disease biology and informing clinical management in DLBCL.

In addition to molecular assays, p53 immunohistochemistry (IHC) is widely used in routine pathological diagnostics. The correlation between *TP53* genetic alterations and p53 protein expression assessed by IHC has been extensively investigated in various tumor types [19,20,21]. In many cancers, missense mutations often lead to accumulation of non-functional but stabilized p53 protein, resulting in strong nuclear staining. In contrast, nonsense and frameshift mutations typically produce truncated or unstable proteins that are rapidly degraded, often resulting in negative p53 staining [22,23,24]. In several solid tumors, p53 IHC has been shown to be a reliable surrogate marker for underlying *TP53* status [21,23,24]. However, in DLBCL, studies evaluating the correlation between p53 IHC and *TP53* genetic alterations are limited, and those available have analyzed mixed cohorts of B-cell lymphomas with inconsistent conclusions. For instance, Lorraine et al. reported poor sensitivity of p53 IHC across various B-cell lymphoma subtypes, suggesting it is not a reliable alternative to molecular testing [25]. In contrast, Xinyi et al. proposed that, in DLBCL, p53 IHC could serve as a potential surrogate marker for *TP53* genetic alterations [26]. Furthermore, the prognostic value of p53 expression in DLBCL remains controversial across studies [27,28,29]. Several studies have evaluated the association between *TP53* alterations, p53 IHC expression, and clinical features or prognosis in DLBCL. Although there is general consensus on the prognostic impact of *TP53* mutations [5,6,10] the prognostic relevance of p53 IHC expression remains controversial [5,27,28,29]. Some studies have reported significant associations between p53 expression and prognosis [5,27,28,29], whereas the other suggest a possible correlation without statistical significance [28,30].

Given the heterogeneity of existing findings, further investigation is warranted using a large cohort to comprehensively evaluate p53 expression, its correlation with *TP53* genetic status, and the prognostic significance in DLBCL.

In this study, we aimed to: (1) comprehensively assess the types and frequencies of *TP53* genetic alterations in DLBCL using both tNGS and OncoScan array; (2) analyze the patterns and interpretation criteria of p53 protein expression using our laboratory p53 IHC LDT; (3) determine the concordance between *TP53* genetic alterations and p53 protein expression to evaluate the clinical utility and diagnostic accuracy of our laboratory p53 IHC LDT as a surrogate for *TP53* mutation status; and (4) investigate the correlation between *TP53* genetic alterations, p53 IHC expression, and clinicopathologic features to assess their prognostic and risk stratification value in DLBCL.

## 2. Methods

### 2.1. Patient Cohort and Sample Collection

**Figure 1:**
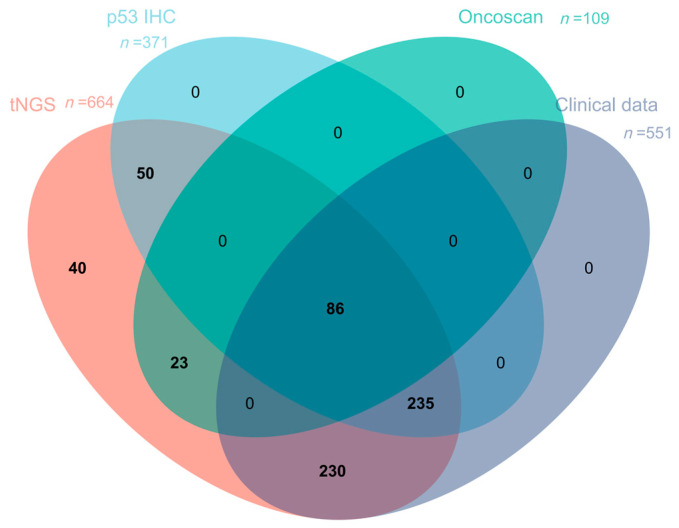
Venn diagram showing case distribution by three detection platforms and clinical data availability. (Legend: This diagram demonstrates the overlap of cases with available datasets for tNGS, p53 immunohistochemistry (IHC), OncoScan array, and clinical data. Intersecting sectors represent case numbers with specific combinations of detectable results. Color coding: tNGS (red), p53 IHC (blue), OnnoScan (green), clinical data (purple)).

A total of 664 patients diagnosed with diffuse large B-cell lymphoma between 2017 and 2023 were retrospectively identified from the Department of Pathology, Fudan University Shanghai Cancer Center. All patients received standard immunochemotherapy (R-CHOP: rituximab, cyclophosphamide, doxorubicin, vincristine, and prednisone; or R-EPOCH: rituximab, etoposide, prednisone, vincristine, cyclophosphamide, and doxorubicin) and underwent tNGS. All 664 DLBCL patients underwent molecular profiling through targeted next-generation sequencing (NGS). Of the total cohort, 371 cases underwent p53 IHC evaluation, while 109 cases were assessed for copy number variations using OncoScan array. From the initial 664 NGS-tested cases, 551 had complete clinical follow-up records available, including 321 with matched IHC results (after excluding primary CNS DLBCL, primary cutaneous DLBCL, primary mediastinal DLBCL, EBV-positive DLBCL, and immunodeficient patients) (Table 1, Figure 1). H&E and IHC slides were independently reviewed by two experienced pathologists. Clinical data, including age, sex, disease stage, and treatment response, were retrieved from medical records.

### 2.2. DNA Extraction and Targeted Next-Generation Sequencing (tNGS)

DNA was isolated from 5-µm-thick formalin-fixed paraffin-embedded (FFPE) tissue sections using the QIAGEN DNA FFPE Tissue Kit (Qiagen, Shanghai, China) in strict accordance with the manufacturer’s recommended protocol. tNGS was performed on all 664 DLBCL samples using the Illumina HiSeq 4000 platform with paired-end sequencing. The average sequencing depth was 1000×. The *TP53* gene was covered from exons 2–11. Variants with a variant allele frequency (VAF) > 5% were retained for analysis. Based on COSMIC, ClinVar, and the *TP53* mutation database, mutations were categorized as benign, likely benign, variants of unknown significance (VUS), likely pathogenic, or pathogenic. Only variants classified as VUS, likely pathogenic, or pathogenic were included in downstream analysis.

### 2.3. OncoScan Copy Number Analysis

Copy number alterations (CNAs) were assessed in 109 NGS-detected cases using the OncoScan^®^ FFPE Assay Kit (Thermo Fisher Scientific, Waltham, MA, USA) following the manufacturer’s instructions. Briefly, 80 ng of FFPE-derived DNA was hybridized with MIP probes, amplified, and scanned using the GeneChip^®^ System (Thermo Fisher Scientific, Santa Clara, CA, USA). Data were processed with OncoScan^®^ Console software (Version 1.3). A region was defined as having a deletion if the following criteria were met: (1) state ≤ 1.33 (including clear deletion [state = 1] and partial subclonal deletion [state = 1.33]); (2) median log2 ratio < −0.3 [31].

### 2.4. Immunohistochemistry (IHC)

IHC staining for p53 was performed on FFPE tissue sections using a monoclonal anti-p53 antibody (DO-7, 1:500; Dako, Carpinteria, CA, USA) (detailed protocols are described in the Supplementary Methods). The intensity and percentage of positive tumor cells were evaluated independently by two pathologists. The average percentage from both observers was used. In the cohort of 371 cases with available FFPE tissue samples analyzed by tNGS and p53 IHC data, ROC curve analysis was performed to determine the optimal cutoff for predicting *TP53* mutation status. A cutoff of 65% demonstrated high sensitivity and specificity and yielded the highest Youden index (0.64), thus being selected as the optimal threshold (AUC = 0.78, 95% CI 0.71–0.85; Supplementary Figure S1, Supplementary Table S1). The immunohistochemical staining intensity for p53 was evaluated using a three-tier scoring system: strong intensity was characterized by dark brown nuclear staining, moderate intensity by distinct brown-yellow nuclear staining, and weak intensity by light brown nuclear staining (Supplementary Figure S2).

Cases were categorized as p53-null when demonstrating nuclear positivity in <1% of tumor cells with nuclear staining at any intensity level, p53-wild-type (p53-wt) when showing 1–65% of tumor cells with nuclear staining at any intensity level, and p53-overexpression (p53-mut) when ≥65% of tumor cells exhibited strong nuclear staining. This classification system was applied consistently by two independent pathologists blinded to the molecular results, with discrepant cases resolved through consensus review. The p53 immunohistochemical staining was performed using a laboratory-developed test (LDT) with non-standardized and unknown analytical sensitivity.

### 2.5. Statistical Analysis

Categorical variables were compared using the chi-square test. Univariate and multivariate survival analyses were conducted using Cox proportional hazards regression. Kaplan–Meier curves were used to assess progression-free survival (PFS). For diagnostic performance evaluation, receiver operating characteristic (ROC) curve analysis was implemented using the pROC package (version 1.18.0) in R, with optimal cutoff determination based on Youden’s index. The visualization of data completeness across different detection methods was achieved through Venn diagrams created with the ggVennDiagram package (version 1.2.2). All statistical tests were two-sided, with *p* < 0.05 considered significant. The complete analytical workflow, including data visualization, was executed in the R statistical environment (version 4.2.2; R Foundation for Statistical Computing).

## 3. Results

### 3.1. TP53 Genetic Alterations Identified by tNGS

**Figure 2:**
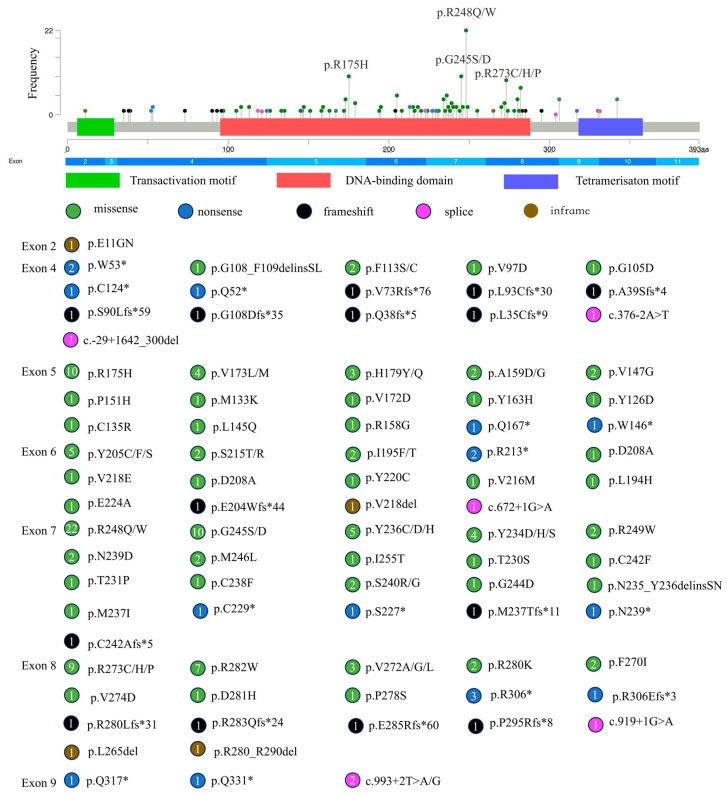
Lollipop plot of *TP53* mutations. The diagram depicts various domains of the *TP53* gene and corresponding amino acid positions. Each “lollipop” represents a mutation at a specific site, with colors indicating different mutation types: green, missense mutations; blue, nonsense mutations; black, frameshift mutations; pink, splice site mutations; brown, inframe indels. The asterisk (*) denotes a premature termination codon as per HGVS nomenclature.

Among 664 DLBCL cases, 170 cases (25.6%) harbored *TP53* alterations, comprising 146 cases with solely mutations, 9 with isolated copy number losses (CNLs), and 15 with concurrent mutations and CNLs. Twenty-eight patients (28/170, 16.5%) harbored compound *TP53* alteration types. In total, 186 *TP53* alterations events in 170 cases were identified, including 161 cases with 177 mutation events primarily located in the DNA-binding domain and 9 cases with only CNLs. The most frequently affected exons were exon 5 (31/177, 17.5%), exon 6 (20/177, 11.3%), exon 7 (62/177, 35.0%), and exon 8 (37/177, 20.9%). Missense mutations were the most common (133/177, 75.2%), followed by nonsense (18/177, 10.2%), frameshift (17/177, 9.6%), splice-site (6/177, 3.4%), and in-frame deletions (3/177, 1.7%). High-frequency hotspot mutations included p.R248Q/W (*n* = 22) and p.R175H (*n* = 10) (Figure 2) (Supplementary Table S2).

### 3.2. TP53 Copy Number Variation (CNV) by OncoScan Array

**Figure 3:**
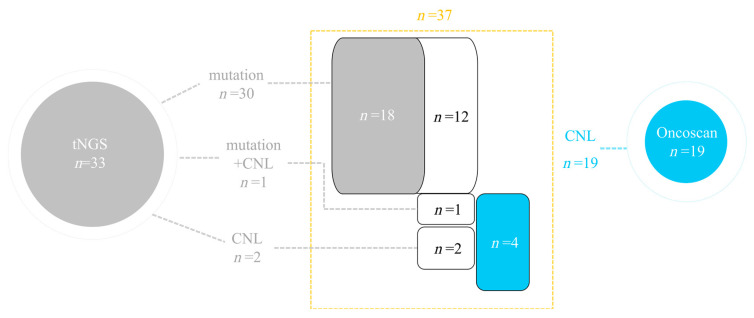
Detection of *TP53* mutations and copy number losses (CNLs) by tNGS and OncoScan array in 109 DLBCL cases. Gray regions indicate alterations detected by tNGS; blue regions indicate copy number loss detected by Oncoscan; white regions indicate overlapping findings detected by both platforms. Yellow box indicates cases with *TP53* alterations among the 109 cases.

CNLs were observed in 19 cases (17.4%, 19/109), of which 13 also carried *TP53* mutations (13/19, 68.4%). Among the six cases with *TP53* CNLs only, two were detected by both tNGS and OncoScan array, while four were identified exclusively by OncoScan array. tNGS can identify the majority of *TP53* genetic alterations (33/37, 89.2%) in DLBCL (Figure 3).

### 3.3. Correlation Between TP53 Genetic Alterations and p53 IHC Expression

**Figure 4:**
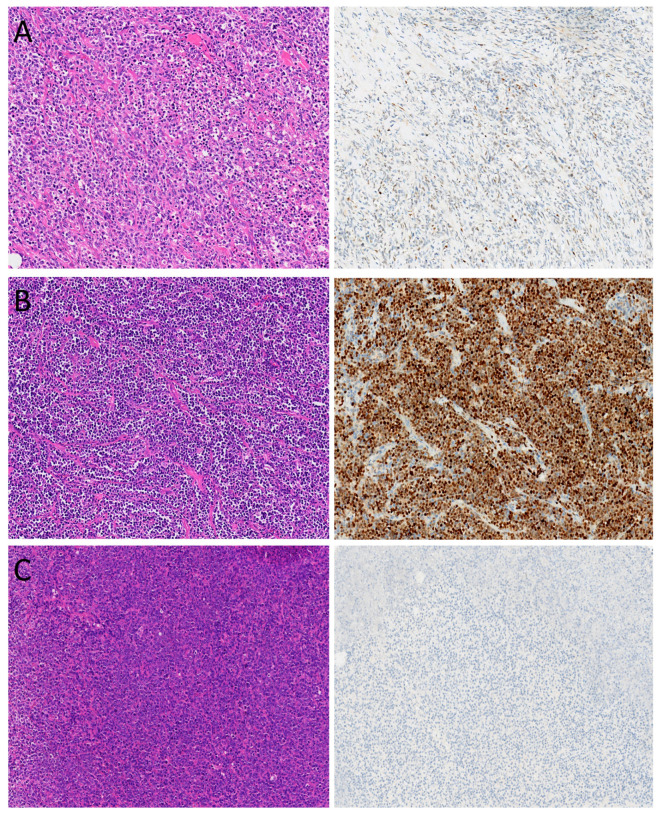
Representative histological images and p53 immunohistochemical staining patterns in DLBCL. (**A**) p53-wt; (**B**) p53-mut; (**C**) p53-null. Original magnification: 400×.

P53 IHC staining localized to the nucleus. Of these 371 cases, 78 (21%) showed diffuse strong positivity (≥65% positive cells, p53-mut), 21 (5.7%) were negative (<1%, p53-null), and 272 (73.3%) showed scattered or focal weak-to-moderate staining (1–65%, p53-wt) (Figure 4) (Table 2).

Among the 96 cases with *TP53* mutations and/or CNLs, 66 (68.8%) were p53-mut, 10 (10.4%) p53-null, and 20 (20.8%) p53-wt. In contrast, of 275 wild-type cases, 12 (4.4%) were p53-mut, 11 (4%) p53-null, and 252 (91.6%) p53-wt. The sensitivity, specificity, and overall accuracy of p53 IHC LDT for detecting *TP53* mutations/CNLs were 79.2%, 91.6%, and 88.4%, respectively.

### 3.4. p53 IHC Expression Across Genetic Alteration Types

Distinct p53 immunohistochemical (IHC) expression patterns were observed across different *TP53* alteration types (including various mutation classes and copy number losses). We therefore systematically analyzed the IHC profiles associated with each genomic variant category. Among 65 cases with missense mutations, 54 (83.1%) were p53-mut and 2 (3.1%) were p53-null. Of seven cases with nonsense mutations, six (85.7%) were p53-wt. Two cases with in-frame deletions were p53-mut. Of six cases with frameshift mutations, four (66.7%) were p53-null. All three splice-site mutations were p53-mut (Table 2, Supplementary Table S3).

In cases with ≥2 alterations, the IHC expression varied depending on mutation type. For example, 6/6 cases with co-occurring CNLs and missense mutations were p53-mut, while 1/2 with CNLs and frameshift were p53-null. These findings indicate that, in cases with concurrent CNLs, the p53 IHC expression profile is primarily consistent with a mutant pattern (Table 2). 

Inconsistencies between IHC and mutation status were mostly observed in cases with missense, nonsense, or frameshift mutations that showed weak to moderate (5–50%) nuclear staining. Cases with discordant *TP53* alterations and p53 expression patterns are shown in Table 3.

### 3.5. Correlation of TP53 Genetic Alterations and p53 IHC with Clinical Features

Among 551 patients with clinical data, 139 had *TP53* mutations/CNLs and 412 were wild type. The cohort included 297 males (53.9%) and 254 females (46.1%), with a median age of 56 years (range 16–82)

Compared to wild type, *TP53*-mutant/CNL cases showed a higher proportion of B symptoms (*p* < 0.01) and higher ECOG score (>2, *p* < 0.05). There were no significant differences in primary site, COO classification, Ann Arbor stage, or extranodal involvement. Complete response (CR) rates were significantly lower in the mutation/CNL group than in the wild-type group (68.3% vs. 82.5%, *p* < 0.01) (Table 4). Similarly, p53-mut/null cases had higher ECOG score and a higher proportion of B symptoms (*p* < 0.05) and worse clinical features and CR rates (66.7% vs. 82.5%, *p* < 0.01) compared to p53-wt, with no significant differences in other clinicopathologic features (Supplementary Table S4).

### 3.6. Prognostic Significance of TP53 Alterations and p53 IHC Expression

Univariate survival analysis (*n* = 551 for *TP53* status; *n* = 321 for p53 IHC) identified *TP53* mutations/CNLs, Ann Arbor stage > 2, extranodal involvement ≥ 2, ECOG ≥ 2, LDH > 250 U/L, B symptoms, IPI > 2, and male as predictors of shorter progression-free survival (PFS).

**Figure 5:**
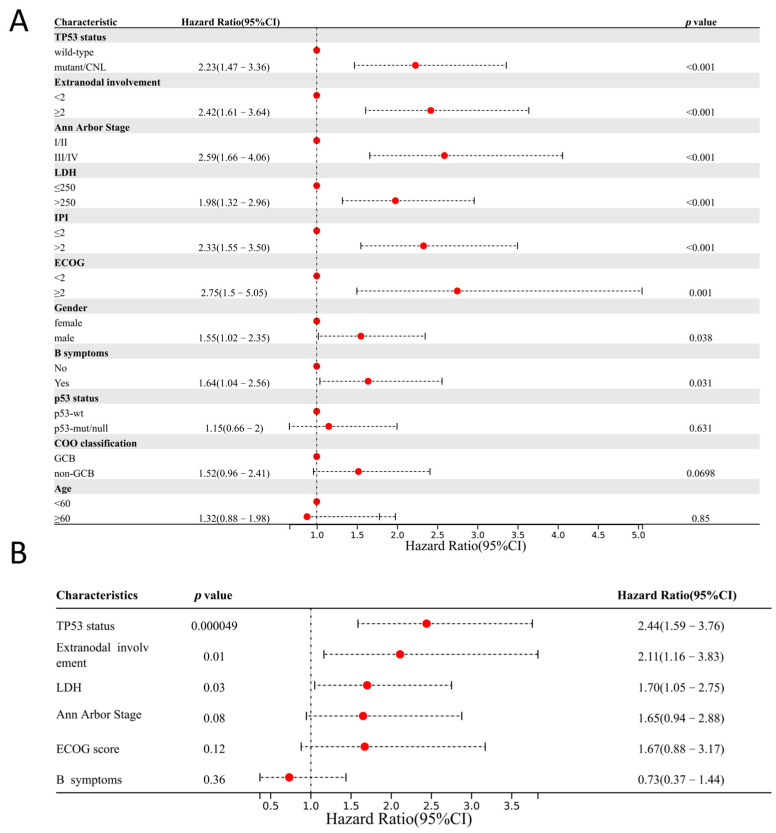
Prognostic factor analysis in DLBCL. (**A**) Univariate Cox regression analysis showing *TP53* mutations/deletions, extranodal involvement ≥ 2, Ann Arbor stage > 2, ECOG ≥ 2, LDH > 250 U/L, IPI > 2, male, and B symptoms as predictors of shorter progression-free survival. (**B**) Multivariate Cox regression analysis identifying *TP53* mutations/deletions, extranodal involvement ≥ 2, and LDH > 250 U/L as independent prognostic factors in DLBCL.

**Figure 6:**
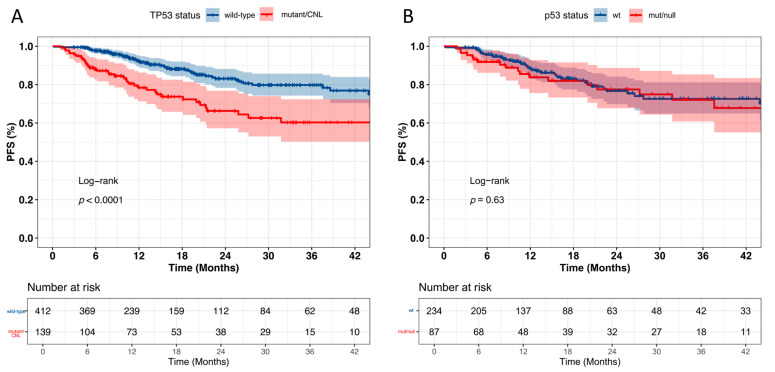
(**A**) *TP53* mutations and/or copy number loss (CNL) (mutant/CNL) associated with shorter PFS. (**B**) No significant correlation between p53 status and PFS.

Multivariate Cox analysis showed that *TP53* status, extranodal involvement ≥ 2, and elevated LDH level were independent adverse prognostic factors. p53 IHC expression, as detected by our LDT assay, was not significantly associated with PFS (Figure 5). Kaplan–Meier analysis demonstrated significantly worse PFS in the *TP53*-mutant group compared to wild type. Although PFS was also lower in the p53-mut/null group than in the p53-wt group, the difference was not statistically significant (Figure 6), highlighting that *TP53* genetic status may be a more reliable prognostic marker in DLBCL (Supplementary Figure S3).

**Figure 7:**
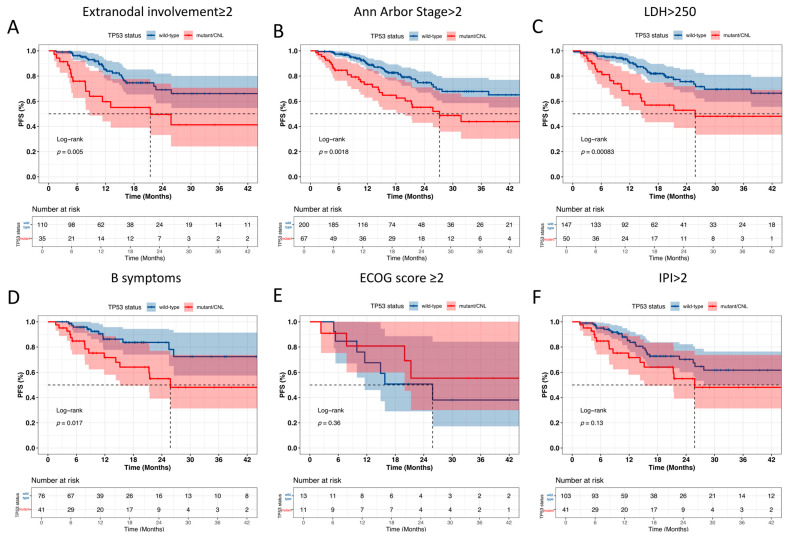
Log-rank analysis of *TP53* and PFS in patients with different risk factors. (**A**–**D**) *TP53* mutant/CNL showed worse prognosis in patients with extranodal involvement ≥ 2, stage > 2, LDH > 250, or B symptoms. (**E**,**F**) No significant prognostic difference between *TP53* mutant/CNL and *TP53* wild-type in patients with ECOG ≥ 2 or IPI > 2.

Subgroup analysis showed that, in patients with extranodal involvement ≥ 2, stage > 2, LDH > 250, or B symptoms, those with *TP53* mutations/CNLs had significantly worse outcomes. However, no significant survival differences were observed in patients stratified by ECOG score ≥ 2 or IPI > 2 (Figure 7).

## 4. Discussion

The *TP53* tumor suppressor gene plays a pivotal role in the pathogenesis of both solid tumors and hematologic malignancies. In DLBCL, *TP53* mutations and CNLs have been linked to aggressive clinical behavior and poor outcomes, as reported in previous studies [5,6]. The LymphGen classification system incorporates *TP53* alterations including both mutations and CNLs into the definition of the A53 subtype, emphasizing their clinical importance [32]. However, the combined use of tNGS and copy number analysis remains resource-intensive, prompting the exploration of IHC for p53 as a cost-effective alternative. Our study systematically evaluated the performance of tNGS, OncoScan, and p53 IHC in detecting *TP53* alterations and revealed critical insights into their diagnostic and prognostic utility.

In this study, tNGS was performed on 664 DLBCL cases, revealing a *TP53* alteration frequency of 25.6%, with mutations accounting for 24.2%. Most mutations were located in the DNA-binding domain and occurred predominantly on exon 5 (17.5%), exon 6 (11.3%), exon 7 (35.0%), and exon 8 (20.9%). Missense mutations were the most frequent, followed by nonsense mutations, consistent with previous reports [6,10,32]. To evaluate the detection capability of tNGS for cases with CNLs, 109 cases underwent additional OncoScan array analysis, which identified 19 cases (17.4%) with *TP53* CNLs. Of these, 13 cases (68.4%) also harbored *TP53* mutations, in agreement with findings from Chapuy et al. [10]. Combining tNGS and OncoScan array results, a total of 37 *TP53*-altered cases were identified among the 109 tested, with tNGS alone detecting 33 cases (89.2%). These findings suggest that tNGS can effectively detect most *TP53* alterations, and only a small proportion of *TP53* CNLs may be missed. Fluorescence in situ hybridization (FISH) or complementary techniques could be considered to further improve detection for *TP53* genetic alterations, which may improve accurate risk stratification.

Although molecular testing has become increasingly integral to modern diagnostics, the combined use of tNGS and copy number analysis is resource-intensive. This has prompted interest in IHC-based assessment of p53 protein expression as a cost-effective alternative. In various solid tumors, p53 IHC demonstrates high sensitivity and specificity [24,33,34]; however, studies evaluating its concordance with *TP53* mutations in DLBCL are limited and yield inconsistent results. Previous studies by Pekka et al. [35] and Lorraine et al. [25] reported sensitivities of 56% and 65.7%, respectively, while the study by Xinyi et al. reported a sensitivity as high as 90%. In our cohort, p53 IHC was performed on 371 DLBCL cases. Based on previous studies, cutoff values for p53 positivity in B-cell lymphomas have varied between 50% and 65%. In our analysis, a threshold of 65% was determined to be optimal using ROC curve analysis, yielding a sensitivity of 79.2%, specificity of 91.6%, and overall accuracy of 88.4%. The reported differences in accuracy (diagnostic sensitivity and specificity) of p53 IHC are at least partly due to variations in the analytical sensitivity and specificity of the IHC LDT assays used across different studies. Because IHC protocols for detecting p53 protein expression are not standardized, ongoing controversy regarding the diagnostic efficiency of p53 IHC is inevitable. In our report, we highlight that our p53 IHC LDT has unknown analytical sensitivity, underscoring the urgent need for standardization in this area.

In addition to the impact of unknown analytical sensitivity on the diagnostic sensitivity of the p53 IHC assay, our study demonstrates that the sensitivity of p53 IHC also varies depending on the type of *TP53* mutation. Missense mutations and in-frame deletions were typically associated with abnormal accumulation of p53 protein [22], with IHC sensitivity reaching 86.2% (56/65). In contrast, nonsense and frameshift mutations generally led to unstable proteins subject to rapid degradation [22]. However, the detection sensitivity of p53 IHC for nonsense and frameshift mutations was markedly limited, with only 38.5% (5/13) of such mutations being identified. These differences arise from both biological variations resulting in heterogenous protein expression and technical aspects of IHC detection. Our protocol shows higher sensitivity for p53 accumulating mutations than degradation-prone variants. While strong, diffuse nuclear staining (≥65% positivity) remains a reliable indicator of underlying *TP53* alterations, wild-type IHC patterns must be interpreted with caution, particularly in high-risk cases. Notably, some *TP53* wild-type cases may also exhibit p53-null staining patterns, further complicating the accurate assessment of *TP53* status. In our cohort, p53-wt cases displayed variable staining intensity, while no cases exhibiting ≥ 65% positivity with only weak-to-moderate staining intensity were observed in this series. The interpretation criteria and clinical significance require further validation through expanded cohort studies. Nevertheless, our results align with prior studies demonstrating p53 IHC’s high specificity even with various non-standardized IHC LDTs with unknown analytical sensitivity and specificity [25,26] but reinforce that p53-wt cannot exclude clinically significant *TP53* defects, especially nonsense/frameshift mutations or copy number losses. Moving forward, protocol standardization and protocol optimization including refinements—such as optimized antibody dilution, adjusted heat-induced epitope retrieval conditions, or incorporation of C-terminal antibodies—may improve detection accuracy.

To further clarify this issue, we analyzed the clinical and pathological features and treatment responses of 551 DLBCL patients, comparing *TP53*-mutant/CNL and wild-type groups. Patients with *TP53* mutation/CNL had a higher incidence of B symptoms and a higher proportion of individuals with ECOG performance status ≥ 2 (*p* < 0.05). Notably, no statistically significant difference was observed in Ann Arbor staging distribution between the two groups, which aligns with previous research findings [5,36,37,38]. Consistent with previous studies, patients with *TP53* mutation/CNL had a significantly lower complete response rate (CRR) compared to the wild-type group (68.3% vs. 82.5%, *p* < 0.01) [5,39]. Similarly, patients in the p53-mut/null group had lower CRR than the p53-wt group (66.7% vs. 82.5%, *p* < 0.05), suggesting that both *TP53* and p53 status correlate with treatment response.

As previously established, *TP53* demonstrates a well-documented association with prognosis in DLBCL [5,6,10], while the prognostic relevance of p53 remains controversial [5,27,28,29,30]. Consistent with prior studies, our survival analyses identified *TP53* genetic status as an independent prognostic factor in DLBCL through multivariate Cox regression. Kaplan–Meier analysis revealed a significant correlation between PFS and *TP53* genetic status. In agreement with findings from Moreno et al. [28] and Jin et al. [30], p53 IHC status, as determined by our LDT assay, demonstrated no significant prognostic correlation, and no statistically significant association was observed between PFS and p53 protein expression patterns. Despite all potential and real differences between the performance of various p53 IHC LDT assays, the limited prognostic predictive power of p53 IHC in DLBCL may primarily stem from its suboptimal detection capability for certain mutation types. The observed heterogeneity in p53 IHC’s prognostic performance across studies likely reflects two key factors: inherent biological variability among DLBCL patients and interlaboratory discrepancies in staining protocols and interpretation criteria, particularly in cutoff value determination. These technical challenges highlight the critical need for standardized testing protocols and rigorously validated interpretation guidelines in clinical practice. In our study, the optimal p53 IHC cutoff was determined by ROC curve analysis.

Notably, *TP53* mutation/CNL maintained their prognostic stratification power even among high-risk patients with adverse clinical features, including extranodal involvement ≥ 2, Ann Arbor stage > II, and elevated LDH levels (>250 U/L). These findings further underscore the critical importance of accurate *TP53* genetic detection and the continued clinical relevance of molecular profiling in DLBCL risk assessment.

This study represents a large cohort assessing the concordance between *TP53* genetic alterations and p53 IHC expression, as well as their prognostic implications in DLBCL. We provide a comprehensive analysis of *TP53* mutation patterns, copy number losses, and protein expression profiles. However, some limitations remain. Not all cases were subjected to copy number analysis, and copy number losses detected by OncoScan array were not confirmed by FISH. Additionally, incomplete clinical and immunohistochemical data were available for some patients, necessitating further data supplementation. Further validation in future studies with more comprehensive datasets is warranted.

In conclusion, tNGS can effectively detect most *TP53* genetic alterations to predict treatment response and prognosis in DLBCL. tNGS may miss a small portion of CNLs, which can be further improved by enhancing the detection capability of NGS and supplementing FISH or OncoScan detection for the accuracy of *TP53* genetic alterations. p53 IHC LDT in our laboratory demonstrated higher sensitivity for missense and in-frame mutations but performs poorly in detecting frameshift and nonsense mutations. Despite its inferior sensitivity and prognostic value compared to direct *TP53* genotyping and the lack of standardization of p53 IHC performance characteristics, previously published studies and our results show evidence that IHC remains a cost-effective, rapid, and widely available method suitable for initial screening in routine diagnostic workflows.

## Figures and Tables

**Table 1:** Distribution of the cases of different testing and their Clinical Data in the DLBCL Cohort.

	tNGS (*n* = 664)	p53 IHC (*n* = 371)	OncoScan (*n* = 109)
p53 IHC	371	--	86
OncoScan	109	86	--
Clinical Data	551	321	109

**Table 2:** p53 immunohistochemical expression patterns in different *TP53* gene alteration statuses in 371 cases.

		p53 Protein Expression Status		
*TP53* Gene Status		p53-Mut (%)	p53-Null (%)	p53-wt (%)
mutant/CNL (*n* = 96)		66 (68.8)	10 (10.4)	20 (20.8)
	missense (*n* = 65)	54 (83.1)	2 (3.1)	9 (13.8)
	nonsense (*n* = 7)	0 (0)	1 (14.3)	6 (85.7)
	frameshift (*n* = 6)	0 (0)	4 (66.7)	2 (23.3)
	in-frame (*n* = 2)	2 (100)	0 (0)	0 (0)
	splice (*n* = 3)	1 (33.3)	2 (66.7)	0(0)
	CNL (*n* = 1)	0 (0)	0 (0)	1 (100)
	CNL + missense (*n* = 6)	6 (100)	0 (0)	0 (0)
	CNL + frameshift (*n* = 2)	0 (0)	1 (50)	1 (50)
	missense + frameshift (*n* = 2)	2 (100)	0 (0)	0 (0)
	missense + nonsense (*n* = 1)	0 (0)	0 (0)	1 (100)
	splice + nonsense (*n* = 1)	1 (100)	0 (0)	0 (0)
wild type (*n* = 275)		12 (4.4)	11 (4)	252 (91.6)
total (*n* = 371)		78 (21)	21 (5.7)	272 (73.3)

CNL: copy number loss.

**Table 3:** Cases with discordance between *TP53* gene alterations and p53 immunohistochemical expression.

	Type	Protein Change	Allele Frequency	p53 IHC
1	missense	p.E11GN	21.33%	10%
2	missense	p.S215T	31.90%	20%
3	missense	p.S215R	31.44%	20%
4	missense	p.V272A	32.85%	30%
5	missense	p.R248Q	15.64%	5%
6	missense	p.R248W	10.40%	10%
7	missense	p.I195T	19.07%	40%
8	missense	p.G245S	33.62%	50%
9	missense	p.S240G	30.03%	30%
10	missense nonsense	p.M246L p.C229*	31.80% 5.17%	10%
11	frameshift	p.V73Rfs*76	49.59%	10%
12	CNL frameshift frameshift	CNL p.A39Sfs*4 p.Q38Kfs*5	54.75% 76.57% 74.30%	5%
13	frameshift	p.R306Efs*3	53.95%	10%
14	nonsense	p.W53*	22.55%	30%
15	nonsense	p.R306*	26.78%	5%
16	nonsense	p.R306*	20.94%	5%
17	nonsense	p.R306*	71.41%	10%
18	nonsense	p.C124*	53.48%	10%
19	nonsense	p.R342*	69.69%	20%
20	CNL	CNL*	53.85%	5%

CNL: copy number loss.

**Table 4:** Clinicopathological characteristics of *TP53*-mutated/copy number loss cases versus *TP53* wild-type cases.

Characteristics	Mutant/CNL (*n* = 139)	Wild Type (*n* = 412)	*p* Value
p53 status			<0.001
p53-mut/null	64 (46.04%)	18 (4.37%)	
p53-wt	16 (11.51%)	223 (54.13%)	
NA	59 (42.45%)	171 (41.50%)	
Gender			0.61
female	61 (43.88%)	193 (46.84%)	
male	78 (56.12%)	219 (53.16%)	
Age			0.11
<60	89 (64.03%)	230 (55.82%)	
≥60	50 (35.97%)	182 (44.18%)	
COO classification			0.16
GCB	59 (42.45%)	143 (34.71%)	
non-GCB	78 (56.12%)	256 (62.13%)	
NA	2 (1.43%)	13 (3.16%)	
Primary site			0.73
extranodal	87 (62.59%)	249 (60.44%)	
nodal	52 (37.41%)	163 (39.56%)	
Extranodal involvement			0.80
<2	104 (74.82%)	301 (73.06%)	
≥2	35 (25.18%)	110 (26.70%)	
NA	0 (0%)	1 (0.24%)	
Ann Arbor Stage			0.96
I/II	72 (51.80%)	210 (50.97%)	
III/IV	67 (48.20%)	201 (48.79%)	
NA	0 (0%)	1 (0.24%)	
LDH			0.99
≤250	88 (63.31%)	265 (64.32%)	
>250	50 (35.97%)	147 (35.68%)	
NA	1 (0.72%)	0 (0%)	
ECOG score			0.04
<2	128 (92.09%)	392 (95.15%)	
≥2	11 (7.91%)	13 (3.15%)	
NA	0 (0%)	7 (1.70%)	
B symptoms			<0.01
Yes	41 (29.50%)	76 (18.45%)	
No	96 (69.06%)	330 (80.09%)	
NA	2 (1.44%)	6 (1.46%)	
IPI			0.35
<2	98 (70.50%)	309 (75.00%)	
≥2	41 (29.50%)	103 (25.00%)	
Therapeutic response			<0.001
CR	95 (68.34%)	340 (82.52%)	
non-CR	44 (31.66%)	72 (17.48%)	

NA, not available.

## Data Availability

The supporting materials are available from the corresponding author upon reasonable request.

## Supplementary Materials

The following supporting information can be downloaded at: https://www.mdpi.com/article/10.3390/curroncol32090488/s1, Figure S1: ROC curve analysis of p53 immunohistochemistry for predicting *TP53* genetic status; Figure S2: Representative images of p53 immunohistochemical (IHC) staining intensities. A. Strong positive staining B. Moderate staining C. Weak staining (Original magnification: ×400); Figure S3: Correlation of clinicopathological factors with progression-free survival (PFS) in DLBCL. A-G. Patients with LDH > 250 U/L, B symptoms, Ann Arbor stage > 2, ECOG ≥ 2, extranodal involvement ≥ 2, IPI ≥ 2 and patients are male had shorter PFS. H-I. No significant correlation between PFS and age or COO classification; Table S1: ROC Curve Analysis Comparing the Predictive Performance of Different p53 IHC Cutoff Values for *TP53* Genetic Status; Table S2: *TP53* Genetic Alterations Profile of 664 DLBCL Cases; Table S3: *TP53* Genetic Alterations and p53 IHC Expression Patterns; Table S4: Clinicopathological Characteristics of p53-mut/null versus p53-wt Patients; Supplementary Method: Immunohistochemistry.

## References

[1] Levine A.J., Oren M. (2009). The first 30 years of p53: Growing ever more complex. Nat. Rev. Cancer.

[2] Levine A.J. (2020). p53: 800 million years of evolution and 40 years of discovery. Nat. Rev. Cancer.

[3] Vousden K.H., Prives C. (2009). Blinded by the Light: The Growing Complexity of p53. Cell.

[4] Olivier M., Hollstein M., Hainaut P. (2010). TP53 mutations in human cancers: Origins, consequences, and clinical use. Cold Spring Harb. Perspect. Biol..

[5] Xu-Monette Z.Y., Wu L., Visco C., Tai Y.C., Tzankov A., Liu W.M., Montes-Moreno S., Dybkaer K., Chiu A., Orazi A. (2012). Mutational profile and prognostic significance of TP53 in diffuse large B-cell lymphoma patients treated with R-CHOP: Report from an International DLBCL Rituximab-CHOP Consortium Program Study. Blood.

[6] Landsburg D.J., Morrissette J.J., Nasta S.D., Barta S.K., Schuster S.J., Svoboda J., Chong E.A., Bagg A. (2023). TP53 mutations predict for poor outcomes in patients with newly diagnosed aggressive B-cell lymphomas in the current era. Blood Adv..

[7] Chiappella A., Diop F., Agostinelli C., Novo M., Nassi L., Evangelista A., Ciccone G., Di Rocco A., Martelli M., Melle F. (2022). Prognostic impact of TP53 mutation in newly diagnosed diffuse large B-cell lymphoma patients treated in the FIL-DLCL04 trial. Br. J. Haematol..

[8] Wen W., Zhang W.L., Tan R., Zhong T.T., Zhang M.R., Fang X.S. (2024). Progress in deciphering the role of p53 in diffuse large B-cell lymphoma: Mechanisms and therapeutic targets. Am. J. Cancer Res..

[9] Schmitz R., Wright G.W., Huang D.W., Johnson C.A., Phelan J.D., Wang J.Q., Roulland S., Kasbekar M., Young R.M., Shaffer A.L. (2018). Genetics and Pathogenesis of Diffuse Large B-Cell Lymphoma. N. Engl. J. Med..

[10] Chapuy B., Stewart C., Dunford A.J., Kim J., Kamburov A., Redd R.A., Lawrence M.S., Roemer M.G.M., Li A.J., Ziepert M. (2018). Molecular subtypes of diffuse large B cell lymphoma are associated with distinct pathogenic mechanisms and outcomes. Nat. Med..

[11] Liu Y., Su Z., Tavana O., Gu W. (2024). Understanding the complexity of p53 in a new era of tumor suppression. Cancer Cell.

[12] Brummer T., Zeiser R. (2024). The role of the MDM2/p53 axis in antitumor immune responses. Blood.

[13] de Leval L., Alizadeh A.A., Bergsagel P.L., Campo E., Davies A., Dogan A., Fitzgibbon J., Horwitz S.M., Melnick A.M., Morice W.G. (2022). Genomic profiling for clinical decision making in lymphoid neoplasms. Blood.

[14] Stiewe T., Haran T.E. (2018). How mutations shape p53 interactions with the genome to promote tumorigenesis and drug resistance. Drug Resist. Updat..

[15] Joerger A.C., Stiewe T., Soussi T. (2025). TP53: The unluckiest of genes?. Cell Death Differ..

[16] Kennedy M.C., Lowe S.W. (2022). Mutant p53: It’s not all one and the same. Cell Death Differ..

[17] Robles A.I., Harris C.C. (2010). Clinical outcomes and correlates of TP53 mutations and cancer. Cold Spring Harb. Perspect. Biol..

[18] Lu T.X., Young K.H., Xu W., Li J.Y. (2016). TP53 dysfunction in diffuse large B-cell lymphoma. Crit. Rev. Oncol. Hematol..

[19] Kim K.M., Ahn A.R., Park H.S., Jang K.Y., Moon W.S., Kang M.J., Ha G.W., Lee M.R., Chung M.J. (2022). Clinical significance of p53 protein expression and TP53 variation status in colorectal cancer. BMC Cancer.

[20] Mueller S., Grote I., Bartels S., Kandt L., Christgen H., Lehmann U., Gluz O., Graeser M., Kates R., Harbeck N. (2023). p53 Expression in Luminal Breast Cancer Correlates with TP53 Mutation and Primary Endocrine Resistance. Mod. Pathol..

[21] Hwang H.J., Nam S.K., Park H., Park Y., Koh J., Na H.Y., Kwak Y., Kim W.H., Lee H.S. (2020). Prediction of TP53 mutations by p53 immunohistochemistry and their prognostic significance in gastric cancer. J. Pathol. Transl. Med..

[22] Guedes L.B., Almutairi F., Haffner M.C., Rajoria G., Liu Z., Klimek S., Zoino R., Yousefi K., Sharma R., De Marzo A.M. (2017). Analytic, Preanalytic, and Clinical Validation of p53 IHC for Detection of TP53 Missense Mutation in Prostate Cancer. Clin. Cancer Res..

[23] Kang E.Y., Cheasley D., LePage C., Wakefield M.J., da Cunha Torres M., Rowley S., Salazar C., Xing Z., Allan P., Bowtell D.D.L. (2021). Refined cut-off for TP53 immunohistochemistry improves prediction of TP53 mutation status in ovarian mucinous tumors: Implications for outcome analyses. Mod. Pathol..

[24] Vermij L., Léon-Castillo A., Singh N., Powell M.E., Edmondson R.J., Genestie C., Khaw P., Pyman J., McLachlin C.M., Ghatage P. (2022). p53 immunohistochemistry in endometrial cancer: Clinical and molecular correlates in the PORTEC-3 trial. Mod. Pathol..

[25] de Haan L.M., de Groen R.A.L., de Groot F.A., Noordenbos T., van Wezel T., van Eijk R., Ruano D., Diepstra A., Koens L., Nicolae-Cristea A. (2024). Real-world routine diagnostic molecular analysis for TP53 mutational status is recommended over p53 immunohistochemistry in B-cell lymphomas. Virchows Arch..

[26] Li X., Luo D., Zhang L., Li Q., Fan J., Zhang J., Huang B., Yang M., Nie X., Chang X. (2023). Accurate interpretation of p53 immunohistochemical patterns is a surrogate biomarker for TP53 alterations in large B-cell lymphoma. BMC Cancer.

[27] Wang X.J., Medeiros L.J., Bueso-Ramos C.E., Tang G., Wang S., Oki Y., Desai P., Khoury J.D., Miranda R.N., Tang Z. (2017). P53 expression correlates with poorer survival and augments the negative prognostic effect of MYC rearrangement, expression or concurrent MYC/BCL2 expression in diffuse large B-cell lymphoma. Mod. Pathol..

[28] Moreno J.C.A., Bahmad H.F., Aljamal A.A., Delgado R., Salami A., Guillot C., Castellano-Sánchez A.A., Medina A.M., Sriganeshan V. (2023). Prognostic Significance of p53 and p63 in Diffuse Large B-Cell Lymphoma: A Single-Institution Experience. Curr. Oncol..

[29] Bouroumeau A., Bussot L., Bonnefoix T., Fournier C., Chapusot C., Casasnovas O., Martin L., McLeer A., Col E., David-Boudet L. (2021). c-MYC and p53 expression highlight starry-sky pattern as a favourable prognostic feature in R-CHOP-treated diffuse large B-cell lymphoma. J. Pathol. Clin. Res..

[30] Jin Y., Wang Y., Wang L., Zhang H., Ren B., Zheng J., Xia Q., Liu Y. (2025). TP53 mutation and immunohistochemical p53 expression characteristics in diffuse large B-cell lymphoma. Front. Oncol..

[31] Van Loo P., Nordgard S.H., Lingjærde O.C., Russnes H.G., Rye I.H., Sun W., Weigman V.J., Marynen P., Zetterberg A., Naume B. (2010). Allele-specific copy number analysis of tumors. Proc. Natl. Acad. Sci. USA.

[32] Wright G.W., Huang D.W., Phelan J.D., Coulibaly Z.A., Roulland S., Young R.M., Wang J.Q., Schmitz R., Morin R.D., Tang J. (2020). A Probabilistic Classification Tool for Genetic Subtypes of Diffuse Large B Cell Lymphoma with Therapeutic Implications. Cancer Cell.

[33] Tessier-Cloutier B., Kortekaas K.E., Thompson E., Pors J., Chen J., Ho J., Prentice L.M., McConechy M.K., Chow C., Proctor L. (2020). Major p53 immunohistochemical patterns in in situ and invasive squamous cell carcinomas of the vulva and correlation with <em>TP53</em> mutation status. Mod. Pathol..

[34] Köbel M., Piskorz A.M., Lee S., Lui S., LePage C., Marass F., Rosenfeld N., Mes Masson A.M., Brenton J.D. (2016). Optimized p53 immunohistochemistry is an accurate predictor of TP53 mutation in ovarian carcinoma. J. Pathol. Clin. Res..

[35] Peroja P., Pedersen M., Mantere T., Nørgaard P., Peltonen J., Haapasaari K.M., Böhm J., Jantunen E., Turpeenniemi-Hujanen T., Rapakko K. (2018). Mutation of TP53, translocation analysis and immunohistochemical expression of MYC, BCL-2 and BCL-6 in patients with DLBCL treated with R-CHOP. Sci. Rep..

[36] Du K.X., Wu Y.F., Hua W., Duan Z.W., Gao R., Liang J.H., Li Y., Yin H., Wu J.Z., Shen H.R. (2024). Identify truly high-risk TP53-mutated diffuse large B cell lymphoma patients and explore the underlying biological mechanisms. Cell Commun. Signal..

[37] Schiefer A.I., Kornauth C., Simonitsch-Klupp I., Skrabs C., Masel E.K., Streubel B., Vanura K., Walter K., Migschitz B., Stoiber D. (2015). Impact of Single or Combined Genomic Alterations of TP53, MYC, and BCL2 on Survival of Patients with Diffuse Large B-Cell Lymphomas: A Retrospective Cohort Study. Medicine.

[38] Dodero A., Guidetti A., Marino F., Tucci A., Barretta F., Re A., Balzarotti M., Carniti C., Monfrini C., Chiappella A. (2022). Dose-adjusted EPOCH and rituximab for the treatment of double expressor and double-hit diffuse large B-cell lymphoma: Impact of TP53 mutations on clinical outcome. Haematologica.

[39] Young K.H., Leroy K., Møller M.B., Colleoni G.W., Sánchez-Beato M., Kerbauy F.R., Haioun C., Eickhoff J.C., Young A.H., Gaulard P. (2008). Structural profiles of TP53 gene mutations predict clinical outcome in diffuse large B-cell lymphoma: An international collaborative study. Blood.

