# Supplementary material for: Correlation of TP53 Genetic Alterations with p53 Immunohistochemical Expression and Their Prognostic Significance in DLBCL
Source: Curr Oncol. 2025 Aug 31;32(9):488. doi: 10.3390/curroncol32090488 (PMC12468183; doi:10.3390/curroncol32090488)
Supplement: Supplementary file 1 [file curroncol-32-00488-s001.zip › Supplementary Figure 2.pdf]

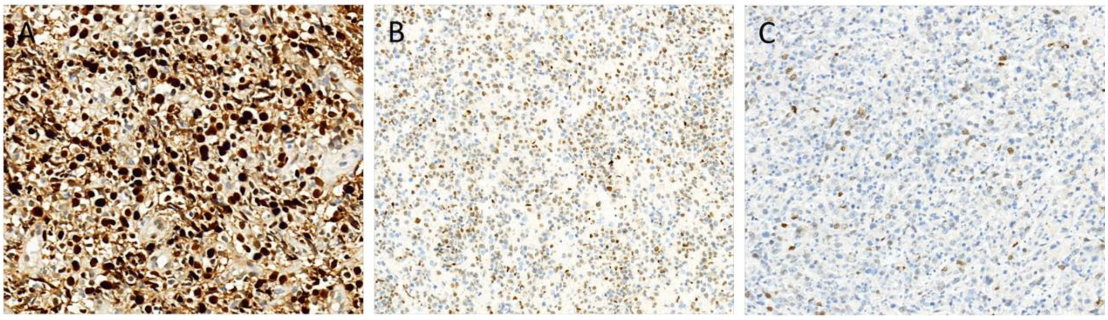

Scheme 2. Representative images of p53 immunohistochemical (IHC) staining intensities. A. Strong positive staining B. Moderate staining C. Weak staining (Original magnification: ×400)
