# Supplementary material for: Correlation of TP53 Genetic Alterations with p53 Immunohistochemical Expression and Their Prognostic Significance in DLBCL
Source: Curr Oncol. 2025 Aug 31;32(9):488. doi: 10.3390/curroncol32090488 (PMC12468183; doi:10.3390/curroncol32090488)
Supplement: Supplementary file 1 [file curroncol-32-00488-s001.zip › Supplementary Figure 3.pdf]

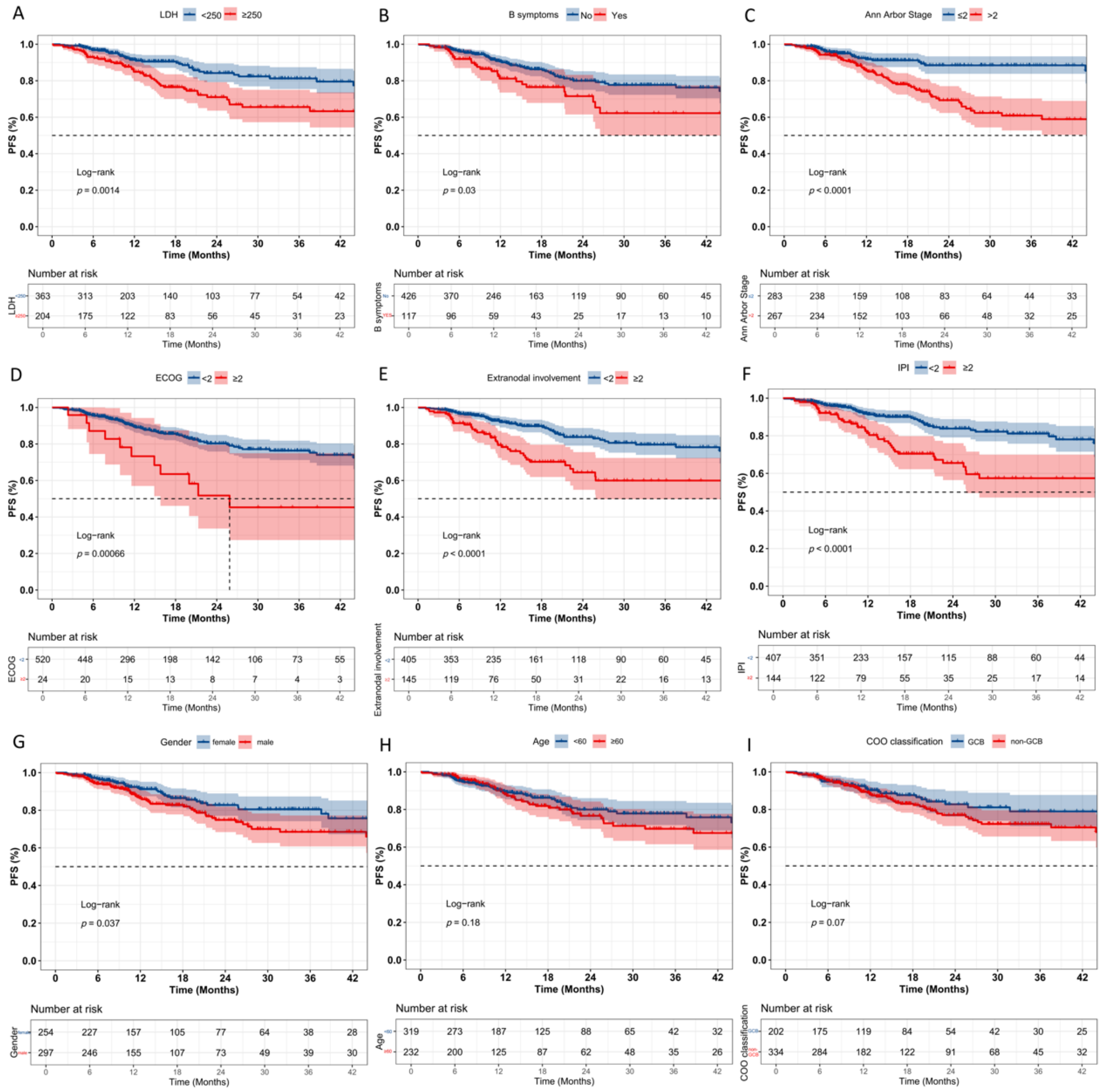

Supplementary Figure 3. Correlation of clinicopathological factors with progression-free survival (PFS) in DLBCL. A-G. Patients with LDH>250 U/L, B symptoms, Ann Arbor stage>2, ECOG≥2, extranodal involvement≥2, IPI≥2 and patients are male had shorter PFS. H-I. No significant correlation between PFS and age or COO classification.
