# Supplementary material for: Correlation of TP53 Genetic Alterations with p53 Immunohistochemical Expression and Their Prognostic Significance in DLBCL
Source: Curr Oncol. 2025 Aug 31;32(9):488. doi: 10.3390/curroncol32090488 (PMC12468183; doi:10.3390/curroncol32090488)
Supplement: Supplementary file 1 [file curroncol-32-00488-s001.zip › Supplementary Method.pdf]

Immunohistochemistry：

Immunohistochemical staining for p53 was performed on formalin-fixed, paraffin-embedded (FFPE) tissue sections using an automated staining system (Ventana Benchmark Ultra, Roche Diagnostics, Switzerland). Briefly, 4-μm-thick tissue sections were deparaffinized and subjected to heat-induced epitope retrieval at 95°C for 36 minutes. The automated protocol included built-in steps for inactivation of endogenous peroxidases and protein blocking using the manufacturer's proprietary reagents. The slides were then incubated with the anti-p53 primary antibody (clone DO-7; Dako, USA; dilution 1:500) at 37°C for 32 minutes. Detection was carried out using the Ventana OptiView DAB Detection Kit at 36°C for 8 minutes. Finally, the sections were counterstained with hematoxylin at 36°C for 8 minutes, followed by bluing reagent at 36°C for 8 minutes to optimize nuclear contrast. Afterward, the slides were dehydrated, cleared, and mounted with a synthetic mounting medium.
