# Supplementary material for: Correlation of TP53 Genetic Alterations with p53 Immunohistochemical Expression and Their Prognostic Significance in DLBCL
Source: Curr Oncol. 2025 Aug 31;32(9):488. doi: 10.3390/curroncol32090488 (PMC12468183; doi:10.3390/curroncol32090488)
Supplement: Supplementary file 1 [file curroncol-32-00488-s001.zip › Supplementary Table 1.pdf]

Supplementary Table 1. ROC Curve Analysis Comparing the Predictive Performance of Different p53 IHC Cutoff Values for *TP53* Genetic Status

| Cutoff | Sensitivity | Specificity | PPV | NPV | Youden index | Optimal |
|---|---|---|---|---|---|---|
| 90% | 30.21% | 100.00% | 100.00% | 80.24% | 0.30 | No |
| 85% | 30.21% | 100.00% | 100.00% | 80.24% | 0.30 | No |
| 80% | 57.29% | 97.79% | 90.16% | 86.64% | 0.55 | No |
| 75% | 57.29% | 97.79% | 90.16% | 86.64% | 0.55 | No |
| 70% | 57.29% | 97.79% | 90.16% | 86.64% | 0.55 | No |
| 65% | 68.75% | 95.59% | 84.62% | 89.66% | 0.64 | Yes |
| 60% | 68.75% | 91.91% | 75.00% | 89.29% | 0.61 | No |
| 55% | 68.75% | 91.91% | 75.00% | 89.29% | 0.61 | No |
| 50% | 69.79% | 88.60% | 68.37% | 89.26% | 0.58 | No |
| 45% | 69.79% | 88.60% | 68.37% | 89.26% | 0.58 | No |
| 40% | 70.83% | 83.09% | 59.65% | 88.98% | 0.54 | No |
| 35% | 70.83% | 83.09% | 59.65% | 88.98% | 0.54 | No |
| 30% | 70.83% | 83.09% | 59.65% | 88.98% | 0.54 | No |
| 25% | 73.96% | 69.85% | 46.41% | 88.37% | 0.44 | No |
