# Supplementary material for: Correlation of TP53 Genetic Alterations with p53 Immunohistochemical Expression and Their Prognostic Significance in DLBCL
Source: Curr Oncol. 2025 Aug 31;32(9):488. doi: 10.3390/curroncol32090488 (PMC12468183; doi:10.3390/curroncol32090488)
Supplement: Supplementary file 1 [file curroncol-32-00488-s001.zip › Supplementary Table 2.pdf]

Supplementary Table 2. *TP53* Genetic Alteration Profile of 664 DLBCL Cases

TP53-status	number (%)
CNL	9(1.36%)
missense	104(15.66%)
splice	5(0.75%)
frameshift	10(1.5%)
nonsense	12(1.82%)
inframe	2(0.30%)
missense+missense	4(0.60%)
cn-del+missense*	11(1.66%)
cn-del+frameshift#	3(0.45%)
cn-del+inframe	1(0.15%)
missense+nonsense	5(0.75%)
missense+frameshift	3(0.45%)
splice+nonsense	1(0.15%)
wild-type	494(74.40%)
total	664(100)

CNL, copy number loss

*Two of the cases had two mutation sites.

#One of the cases had two mutation sites.
