# Supplementary material for: Correlation of TP53 Genetic Alterations with p53 Immunohistochemical Expression and Their Prognostic Significance in DLBCL
Source: Curr Oncol. 2025 Aug 31;32(9):488. doi: 10.3390/curroncol32090488 (PMC12468183; doi:10.3390/curroncol32090488)
Supplement: Supplementary file 1 [file curroncol-32-00488-s001.zip › Supplementary Table 3.pdf]

Supplementary Table 3.*TP53* Genetic Alterations and p53 IHC Expression Patterns

Sample ID	p53 IHC	Mutation Type	Description
1	80%	missense variant	p.I255T
2	0%	splice acceptor variant	c.-29+1642 300del
3	90%	missense variant	p.P151H
4	90%	missense variant	p.R175H
5	0%	splice donor variant	c.672+1G>A
6	90%	missense variant	p.R175H
7	90%	missense variant	p.Y236H
8	10%	missense variant	p.E11GN
9	90%	missense variant	p.A159D
10	90%	missense variant	p.R273C
11	90%	missense variant	p.R273H
12	80%	missense variant	p.Y220C
13	10%	frameshift variant	p.R306Efs*3
14	90%	missense variant	p.V147G
15	70%	missense variant	p.R248Q
16	80%	missense variant	p.R248W
17	90%	missense variant	p.Y205S
18	80%	missense variant	p.R248Q
19	30%	stop gained	p.W53*
20	70%	missense variant	p.G245S
21	70%	missense variant	p.Y234H
22	90%	missense variant	p.R282W
23	80%	missense variant	p.R248Q
24	90%	missense variant	p.R248Q
25	90%	missense variant	p.R248Q
26	5%	CNL	CNL
27	70%	missense variant	p.R248W
28	90%	missense variant	p.R248Q
29	70%	missense variant	p.G245S
30	90%	missense variant	p.R282W
31	80%	missense variant	p.C238F
32	80%	inframe deletion	p.R280 R290del
33	90%	missense variant	p.R175H
34	70%	missense variant	p.S240R
35	80%	missense variant	p.G108 F109delinsSL
36	80%	inframe deletion	p.V218del
37	90%	missense variant	p.R273P

38	95%	missense variant	p.F113S
39	80%	missense variant	p.V172D
40	80%	missense variant	p.R175H
41	20%	stop gained	p.R342*
42	0	stop gained	p.Q167*
43	100%	missense variant	p.R280K
44	5%	stop gained	p.R306*
45	5%	stop gained	p.R306*
46	10%	stop gained	p.C124*
47	80%	missense variant	p.G245D
48	90%	missense variant	p.H179Y
49	20%	missense variant	p.S215T
50	80%	missense variant	p.R249W
51	90%	missense variant	p.G245D
52	0	frameshift variant	p.N239*
53	80%	missense variant	p.R273C
54	70%	missense variant	p.V274D
55	70%	missense variant	p.G245S
56	20%	missense variant	p.S215R
57	80%	missense variant	p.D208A
58	90%	missense variant	p.N239D
59	30%	missense variant	p.V272A
60	10%	frameshift variant	p.V73Rfs*76
61	90%	splice acceptor variant	c.376-2A>T
62	80%	missense variant	p.V218E
63	0	frameshift variant	p.R283Qfs*24
64	80%	missense variant	p.R158G
65	5%	stop gained	p.R306*
66	70%	missense variant	p.F113C
67	90%	missense variant	p.R248Q
68	50%	missense variant	p.G245S
69	30%	missense variant	p.S240G
70	80%	missense variant	p.R248Q
71	90%	missense variant	p.M237I
72	80%	missense variant	p.R273H
73	90%	missense variant	p.R248Q
74	0	frameshift variant	p.C242Afs*5
75	80%	missense variant	p.Y236C
76	0	frameshift variant	p.E285Rfs*60

77	0	missense variant	p.Y205S
78	0	missense variant	p.R248W
79	5%	missense variant	p.R248Q
80	10%	missense variant	p.R248W
81	40%	missense variant	p.I195T
82	80%	missense variant	p.R248Q
		missense variant	p.T231P
83	80%	missense variant	p.E224A
		missense variant	p.Y236H
84	80%	frameshift variant	p.L35Cfs*9
		missense variant	p.N235 Y236delinsSN
85	10%	missense variant	p.M246L
		stop gained	p.C229*
86	90%	splice donor variant	c.993+2T>G
		stop gained	p.Q331*
87	90%	CNL	CNL
		missense variant	p.H179Q
		CNL	CNL
88	80%	missense variant	p.G244D
		missense variant	p.Y236D
89	0	CNL	CNL
		frameshift variant	p.M237Tfs*11
90	80%	missense variant	p.V97D
		missense variant	p.R282W
91	70%	CNL	CNL
		missense variant	p.D281H
92	90%	CNL	CNL
		missense variant	p.P278S
93	80%	CNL	CNL
		missense variant	p.Y205F
94	70%	frameshift variant	p.L93Cfs*30
		missense variant	p.N239D
		CNL	CNL
95	5%	frameshift variant	p.A39Sfs*4
		frameshift variant	p.Q38Kfs*5
		missense variant	p.L145Q
	90%	CNL	CNL
96		missense variant	p.Y236C

CNL: copy number loss
