# Supplementary material for: Correlation of TP53 Genetic Alterations with p53 Immunohistochemical Expression and Their Prognostic Significance in DLBCL
Source: Curr Oncol. 2025 Aug 31;32(9):488. doi: 10.3390/curroncol32090488 (PMC12468183; doi:10.3390/curroncol32090488)
Supplement: Supplementary file 1 [file curroncol-32-00488-s001.zip › Supplementary Table 4.pdf]

Supplementary Table 4. Clinicopathological Characteristics of p53-mut/null versus p53-wt Patients

Characteristics	p53-mut/null(N=87)	p53-wt(N=234)	*p* value
**Gender**			0.85
female	40(45.98%)	103(44.02%)	
male	47(54.02%)	131(55.98%)	
**Age**			0.29
<60	54(62.07%)	128(54.70%)	
≥60	33(37.93%)	106(45.30%)	
**COO classification**			0.68
GCB	37(42.53%)	91(38.89%)	
non-GCB	50(57.47%)	141(60.25%)	
NA	0(0%)	2(0.86%)	
**Primary site**			0.85
Nodal	37(42.53%)	95(40.60%)	
extronodal	50(57.47%)	139(59.40%)	
**Extranodal involvement**			0.76
<2	65(74.71%)	162(69.23%)	
≥2	22(25.29%)	72(30.77%)	
**Ann Arbor Stage**			0.44
I/II	39(44.83%)	118(50.43%)	
III/IV	48(55.17%)	116(49.57%)	
**LDH**			0.46
≤250	51(58.62%)	149(63.68%)	
>250	36(41.38%)	84(35.90%)	

NA	0(0%)	1(0.43%)	
**ECOG score**			0.01
<2	79(90.8%)	226(96.58%)	
≥2	8(9.2%)	5(2.14%)	
NA	0(0%)	3(1.28%)	
**B symptoms**			0.04
No	57(65.52%)	184(78.63%)	
Yes	27(31.03%)	46(19.67%)	
NA	3(3.45%)	4(1.7%)	
**IPI**			0.48
≤2	59(67.82%)	170(72.65%)	
>2	28(32.18%)	64(27.35%)	
NA	0(0%)	0(0%)	
**Therapeutic response**			0.004
CR	58(66.67%)	193(82.48%)	
non-CR	29(33.33%)	41(17.52%)	

NA, not available
